# Ferromagnetic Fluctuations in the Heavily Overdoped Regime of Single-Layer High-*T*_c_ Cuprate Superconductors

**DOI:** 10.3390/ma16217048

**Published:** 2023-11-06

**Authors:** Tadashi Adachi, Koshi Kurashima, Takayuki Kawamata, Takashi Noji, Satoru Nakajima, Yoji Koike

**Affiliations:** 1 Department of Engineering and Applied Sciences, Sophia University, 7-1 Kioi-cho, Chiyoda-ku, Tokyo 102-8554, Japan; 2 Department of Applied Physics, Tohoku University, 6-6-05 Aoba, Aramaki, Aoba-ku, Sendai 980-8579, Japanykoike52@gmail.com (Y.K.); 3 Department of Natural Sciences, Tokyo Denki University, 5 Senju Asahi-cho, Adachi-ku, Tokyo 120-8551, Japan; 4 Center for Liberal Arts and Sciences, Iwate Medical University, 1-1-1 Idai-dori, Yahaba-cho, Shiwa-gun, Iwate 028-3694, Japan

**Keywords:** ferromagnetic fluctuation, Tl-2201 cuprate, La-214 cuprate, Bi-2201 cuprate, electrical resistivity, magnetization

## Abstract

To investigate proposed ferromagnetic fluctuations in the so-called single-layer Bi-2201 and La-214 high-*T*_c_ cuprates, we performed magnetization and electrical resistivity measurements using single-layer Tl-2201 cuprates Tl_2_Ba_2_CuO_6+δ_ and La-214 La_2−_*_x_*Sr*_x_*CuO_4_ in the heavily overdoped regime. Magnetization of Tl_2_Ba_2_CuO_6+δ_ and La_2−_*_x_*Sr*_x_*CuO_4_ exhibited the tendency to be saturated in high magnetic fields at low temperatures, suggesting the precursor behavior toward the formation of a ferromagnetic order. It was found that the power of temperature *n* obtained from the temperature dependence of the electrical resistivity is ~4/3 and ~5/3 for Bi-2201 and La_2−_*_x_*Sr*_x_*CuO_4_, respectively, and is ~4/3 at high temperatures and ~5/3 at low temperatures in Tl_2_Ba_2_CuO_6+δ_. These results suggest that two- and three-dimensional ferromagnetic fluctuations exist in Bi-2201 and La_2−_*_x_*Sr*_x_*CuO_4_, respectively. In Tl_2_Ba_2_CuO_6+δ_, it is suggested that the dimension of ferromagnetic fluctuations is two at high temperatures and three at low temperatures, respectively. The dimensionality of ferromagnetic fluctuations is understood in terms of the dimensionality of the crystal structure and the bonding of atoms in the blocking layer.

## 1. Introduction

In the study of high-*T*_c_ cuprate superconductivity, it is important to clarify the link between the magnetism and superconductivity, and therefore, huge amounts of study have been performed [1]. In the hole-doped cuprates, the parent compound without carrier doping is an antiferromagnetic (AF) Mott insulator, and a small amount of hole doping causes the AF order to disappear and superconductivity to emerge. AF spin fluctuations have been observed in the hole concentration regime where superconductivity appears, and an idea is that AF fluctuations are related to the formation of superconducting electron pairs. In the so-called La-214 cuprates, the charge-spin stripe order and its fluctuations have also been observed [2]. In other cuprates, the charge order and its fluctuations have been observed from recent X-ray scattering and NMR experiments, and the relation to the so-called pseudogap has been discussed [3].

In the overdoped regime, an anomalous metallic state, as well as the disappearance of the pseudogap, has been observed [4]. Moreover, the disappearance of the stripe fluctuations [5] and a phase separation into superconducting and normal-state regions [6,7,8] have also been suggested. For spin fluctuations, neutron scattering experiments in the La-214 cuprate La_2−_*_x_*Sr*_x_*CuO_4_ (LSCO) have revealed that AF fluctuations weaken with overdoping and disappear together with superconductivity, suggesting the close relationship between AF fluctuations and superconductivity [9]. On the other hand, resonant inelastic X-ray scattering (RIXS) experiments using LSCO thin films have reported robust AF fluctuations in the non-superconducting heavily overdoped regime [10]. These indicate that the relationship between superconductivity and AF fluctuations is yet to be solved.

Formerly, it has been proposed from theories [11,12] and experiments [13,14,15] that ferromagnetic orders/fluctuations exist in the heavily overdoped regime and are related to the suppression of superconductivity. Calculations by Teranishi et al. using the FLEX approximation with the one-band Hubbard model have suggested that the spin susceptibility approaches *q* = (0,0) in the heavily overdoped regime [16]. It has been suggested in calculations of the four-band d-p model by Watanabe et al. that the magnetic moment is enhanced in the heavily overdoped regime [17]. Experimentally, measurements of muon spin relaxation (μSR) and the ab-plane electrical resistivity *ρ*_ab_ in heavily overdoped LSCO conducted by Sonier et al. [13] have revealed the enhancement of spin fluctuations and the behavior of the resistivity characteristic of three-dimensional ferromagnetic fluctuations via itinerant electrons, according to the self-consistent renormalization (SCR) theory [18]. We have also measured *ρ*_ab_, magnetization, specific heat, and μSR in the single-layer Bi-2201 cuprate (Bi,Pb)_2_Sr_2_CuO_6+δ_ [14] and found that spin fluctuations are enhanced at low temperatures, and the magnetization curve saturates at low temperatures in high magnetic fields. Moreover, *ρ*_ab_, specific heat, and magnetic susceptibility have shown characteristic behavior of two-dimensional ferromagnetic fluctuations [18]. In Fe-substituted Bi-2201 cuprate Bi_1.74_Pb_0.38_Sr_1.88_Cu_1−_*_y_*Fe*_y_*O_6+δ_, we have observed from magnetization measurements that ferromagnetic fluctuations are enhanced by the Fe substitution [19]. RIXS measurements in Bi-2201 have reported the enhancement of spin fluctuations at *q* ~ (0,0) in the heavily overdoped regime [20]. These results suggest that two-dimensional ferromagnetic fluctuations exist in heavily overdoped Bi-2201. In the electron-doped La_2−_*_x_*Ce*_x_*CuO_4_ in the heavily overdoped regime, the formation of a ferromagnetic order is suggested from measurements of the resistivity, magnetization, polar Kerr effect, etc. [15].

To gain insight into the difference in the dimensionality of ferromagnetic fluctuations between LSCO and Bi-2201, we investigated the single-layer Tl-2201 cuprate Tl_2_Ba_2_CuO_6+δ_, in which the non-superconducting heavily overdoped regime is accessible and the CuO_2_ plane is rather flat. The electrical resistivity and magnetization were measured in heavily overdoped Tl-2201 as well as in heavily overdoped LSCO for comparison.

## 2. Experimental

Polycrystals of Tl_2_Ba_2_CuO_6+δ_ and single crystals of LSCO were prepared by the solid-state reaction [21] and floating zone [22] method, respectively. The obtained samples were confirmed to be of the single phase by X-ray diffraction analysis. The single domain structure of LSCO crystals was confirmed by the X-ray back-Laue photography. For Tl_2_Ba_2_CuO_6+δ_, annealing was performed in 1–3 atm oxygen or 1 atm Ar flow to control the hole concentration. LSCO single crystals were annealed in 1 atm oxygen at 900 °C for 50 h, followed by annealing at 500 °C for 50 h to compensate for the oxygen deficiency [23].

The Sr concentration of LSCO was found to be *x* = 0.29 from inductively coupled plasma analysis. The oxygen deficiency *d* of LSCO estimated from iodometric titration was found to be *d* = 0.005(10) in La_2-*x*_Sr*_x_*CuO_4-*d*_, indicating our LSCO crystals were almost stoichiometric. The hole concentration of Tl_2_Ba_2_CuO_6+δ_ was determined from the empirical formula for the superconducting transition temperature, *T*_c_, and hole concentration per Cu, *p*, by Presland et al. [24]. The electrical resistivity was measured by the dc four-probe method, and the magnetization was measured using a superconducting quantum interference device magnetometer (Quantum Design (Tokyo, Japan), MPMS) in magnetic fields between ±5 T.

## 3. Results

**Figure 1:**
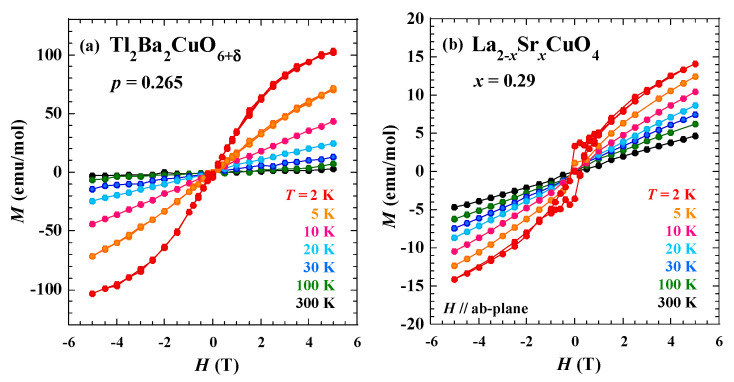
Magnetization curves of heavily overdoped (**a**) Tl_2_Ba_2_CuO_6+δ_ (*p* = 0.265) and (**b**) La_2−_*_x_*Sr*_x_*CuO_4_ (*x* = 0.29).

**Figure 2:**
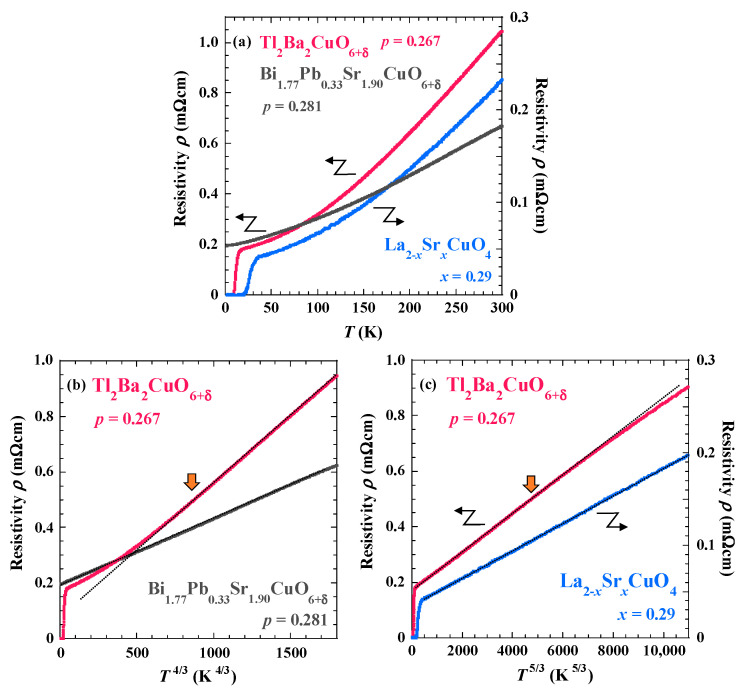
Temperature dependence of the electrical resistivity plotted against (**a**) *T*, (**b**) *T*^4/3^, and (**c**) *T*^5/3^ in heavily overdoped Tl_2_Ba_2_CuO_6+δ_ (*p* = 0.265), La_2−_*_x_*Sr*_x_*CuO_4_ (*x* = 0.29), and Bi_1.77_Pb_0.33_Sr_1.90_CuO_6+δ_ (*p* = 0.281) [14]. Orange arrows in (**b**,**c**) indicate the temperature where the resistivity deviates from the linear relation between the resistivity and *T*^4/3^ (*T*^5/3^). Note that the resistivity of La_2−_*_x_*Sr*_x_*CuO_4_ and Bi_1.77_Pb_0.33_Sr_1.90_CuO_6+δ_ is the ab-plane resistivity *ρ*_ab_.

Figure 1a shows the magnetization curves of Tl_2_Ba_2_CuO_6+δ_ with *p* = 0.265 in the heavily overdoped regime (*T*_c_ ~ 6 K). Above 10 K, the magnetization exhibits paramagnetic behavior (linear in the magnetic field). The slope of magnetization increases with decreasing temperature, in agreement with the previous results [25], where the magnetic susceptibility exhibits a Curie-like behavior. It is found that the magnetization is slightly curved in the high-field region at 5 K, and it tends to saturate in the high-field region at 2 K. A tiny hysteresis behavior is observed between the increasing and decreasing magnetic field, around the zero field at 2 K. This might originate from the pinning of the superconducting vortices, tiny ferromagnetic ordered regions, or others. The overall behavior of the magnetization curve in LSCO with *x* = 0.29, shown in Figure 1b, is almost identical to that in Tl_2_Ba_2_CuO_6+δ_. At 2K, however, a hysteresis behavior in low fields is apparent, which may be due to weak superconductivity, as observed in the resistivity in Figure 2a. The tendency of the saturation of the magnetization in heavily overdoped Tl_2_Ba_2_CuO_6+δ_ and LSCO is similar to those observed in heavily overdoped Bi-2201 [14] and probably indicates the enhancement of ferromagnetic fluctuations.

Figure 2a shows the temperature dependence of the electrical resistivity of Tl_2_Ba_2_CuO_6+δ_ with *p* = 0.265 and LSCO with *x* = 0.29, as well as heavily overdoped Bi-2201 [14]. The superconducting transition is observed at low temperatures in Tl_2_Ba_2_CuO_6+δ_. For LSCO, the superconducting transition is also observed at low temperatures, which may be due to the slightly non-uniform Sr distribution in the crystal. Figure 2b shows the temperature dependence of the electrical resistivity, plotted as the resistivity vs. *T*^4/3^, for Tl_2_Ba_2_CuO_6+δ_ as well as our previously reported heavily overdoped Bi-2201 single crystal [14]. In heavily overdoped Bi-2201, *ρ*_ab_ follows *T*^4/3^ in a wide temperature range below room temperature, suggesting the enhancement of two-dimensional ferromagnetic fluctuations. In Tl_2_Ba_2_CuO_6+δ_, the behavior of resistivity is proportional to *T*^4/3^ above ~160 K, shown by an arrow, and the resistivity deviates upwards from the linear relation between the resistivity and *T*^4/3^ below ~160 K.

A plot of the temperature dependence of the resistivity of Tl_2_Ba_2_CuO_6+δ_ on *T*^5/3^ is shown in Figure 2c, together with the results of LSCO with *x* = 0.29. The *ρ*_ab_ of LSCO is proportional to *T*^5/3^ in a wide temperature range above *T*_c_, while for LSCO with *x* = 0.33, Fermi-liquid behavior proportional to *T*^2^ is observed below 50 K [13]. The difference in the temperature range of *T*^5/3^ may be related to the hole concentration. It is found that the resistivity of Tl_2_Ba_2_CuO_6+δ_ is proportional to *T*^5/3^ below ~160 K down to *T*_c_. These results are summarized as *ρ*_ab_ being proportional to *T*^4/3^ for Bi-2201 and being proportional to *T*^5/3^ for LSCO in a wide temperature range, while the temperature dependence of the resistivity changes between *T*^4/3^ and *T*^5/3^ around 160 K for Tl_2_Ba_2_CuO_6+δ_.

**Figure 3:**
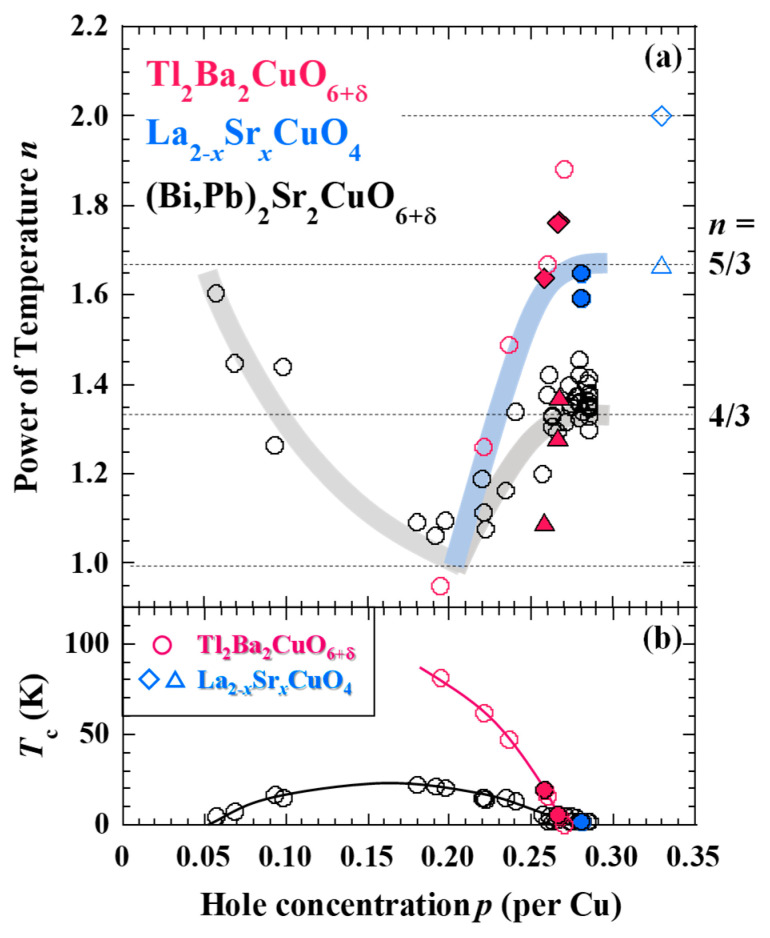
Hole-concentration *p* dependence of (**a**) the power of temperature *n* of *ρ* = A + B*T^n^* and (**b**) *T*_c_ in Tl_2_Ba_2_CuO_6+δ_ and La_2−_*_x_*Sr*_x_*CuO_4_. Solid lines are to guide the reader’s eye. Open symbols are preceding results of Tl_2_Ba_2_CuO_6+δ_ [25], La_2−_*_x_*Sr*_x_*CuO_4_ [13], and (Bi,Pb)_2_Sr_2_CuO_6+δ_ [14]. In (**a**), circles indicate *n* obtained by fitting in a wide temperature range, while diamonds and squares indicate *n* obtained by fitting at low and high temperatures, respectively.

The temperature dependence of the resistivity was fitted by the equation *ρ* = A + B*T^n^* to estimate the power of temperature *n*. The dependencies of *n* and *T*_c_ on the hole concentration in Tl_2_Ba_2_CuO_6+δ_ and LSCO are shown in Figure 3, together with our former results of Bi-2201 [14] and preceding results of Tl_2_Ba_2_CuO_6+δ_ [25] and LSCO [13]. For Tl_2_Ba_2_CuO_6+δ_, both *n* values obtained above and below ~160 K are shown. For the preceding LSCO [13], both *n* values obtained at high and low temperatures are shown. Circles indicate the *n* values obtained by fitting the entire temperature range. For Tl_2_Ba_2_CuO_6+δ_, *n* obtained above ~160 K is close to 4/3 in the heavily overdoped regime, which is similar to that of Bi-2201. On the other hand, *n* below ~160 K is close to 5/3. For LSCO with *x* = 0.29, *n* ~ 5/3, in agreement with that for *x* = 0.33 at high temperatures [13].

## 4. Discussion

The magnetization curves in heavily overdoped Tl_2_Ba_2_CuO_6+δ_ and LSCO showed that the magnetization tended to saturate at low temperatures in high magnetic fields. Such behavior has also been observed in heavily overdoped Bi-2201 [14] and LSCO [13]. Therefore, it is likely that ferromagnetic fluctuations also develop at low temperatures in Tl_2_Ba_2_CuO_6+δ_, which is consistent with the theoretical indication [11]. The SCR theory suggests that *n* = 4/3 is characteristic of two-dimensional ferromagnetic fluctuations and *n* = 5/3 of three-dimensional ferromagnetic fluctuations [18]. Therefore, in the heavily overdoped regime, it is likely that three-dimensional ferromagnetic fluctuations exist in LSCO and two-dimensional ferromagnetic fluctuations in Bi-2201. For heavily overdoped Tl_2_Ba_2_CuO_6+δ_, it is likely that two-dimensional ferromagnetic fluctuations exist at high temperatures and three-dimensional fluctuations at low temperatures.

The difference in the dimensionality of ferromagnetic fluctuations in Tl_2_Ba_2_CuO_6+δ_, LSCO, and Bi-2201 may originate from the difference in the strength of the spin correlation of itinerant electrons between the CuO_2_ planes. As the strength of the spin correlation perpendicular to the CuO_2_ plane is strongly influenced by the crystal structure, the longer the distance between the CuO_2_ planes, the more likely it is that two-dimensional ferromagnetic fluctuations will develop. The interplane distances between the CuO_2_ planes of Bi-2201, Tl_2_Ba_2_CuO_6+δ_, and LSCO are about 12.3 Å, 11.6 Å, and 6.6 Å, respectively. The present results indicate that the temperature range where three-dimensional ferromagnetic fluctuations are observed is wider with the shorter interplane distance between the CuO_2_ plane. The reason for the difference in the dimensionality of the ferromagnetic fluctuations developed in Bi-2201 and Tl_2_Ba_2_CuO_6+δ_ may also be related to the difference in the bonding of atoms in the blocking layer. In Bi-2201, due to the lone pair of electrons of Bi in the blocking layer, two overlapping BiO planes are electrically repulsive, so that the BiO planes are weakly coupled via van der Waals force. On the other hand, it is known in Tl_2_Ba_2_CuO_6+δ_ that the TlO planes are covalently bonded with each other and the LaO planes in LSCO are bonded with each other via ionic coupling. It is therefore likely that the ferromagnetic correlation crossovers from two to three dimensions with decreasing temperature in Tl_2_Ba_2_CuO_6+δ_ owing to the coupling between the CuO_2_ planes being stronger at low temperatures, while they are very two-dimensional in Bi-2201 over a wide temperature range.

Anomalous metallic states have been proposed in the overdoped regime of single-layer copper oxides. In the resistivity of LSCO [26] and Tl_2_Ba_2_CuO_6+δ_ [27], it has been suggested that the T linear component observed near the optimally doped regime extends into the overdoped regime and coexists with the Fermi-liquid *T*^2^ component. It is a future issue to clarify how they connect and whether they are compatible with the characteristic behavior of ferromagnetic fluctuations in the heavily overdoped cuprates.

## 5. Summary

Magnetization curves and electrical resistivity of single-layer Tl_2_Ba_2_CuO_6+δ_, LSCO, and Bi-2201 cuprates in the heavily overdoped regime were measured. The magnetization revealed a tendency to saturate at low temperatures in high magnetic fields, which is considered to be a precursor phenomenon to the formation of a ferromagnetic order. The power of temperature *n* obtained by fitting the temperature dependence of the electrical resistivity was found to be ~4/3 for Bi-2201 and ~5/3 for LSCO. These results suggest the enhancement of two-dimensional and three-dimensional ferromagnetic fluctuations in Bi-2201 and LSCO, respectively. In Tl_2_Ba_2_CuO_6+δ_, *n* ~ 4/3 at high temperatures and *n* ~ 5/3 at low temperatures, suggesting that two-dimensional ferromagnetic fluctuations at high temperatures crossover to three-dimensional ones at low temperatures. The differences in dimensionality may be understood in terms of the dimensionality of the crystal structure and bonding strength of atoms in the blocking layer. It is concluded that ferromagnetic fluctuations are universal in the heavily overdoped regime of the single-layer cuprates.

## Data Availability

No new data were created.
